# Effects of a high fat diet on gut microbiome dysbiosis in a mouse model of Gulf War Illness

**DOI:** 10.1038/s41598-020-66833-w

**Published:** 2020-06-12

**Authors:** Mariana Angoa-Pérez, Branislava Zagorac, Dina M. Francescutti, Andrew D. Winters, Jonathan M. Greenberg, Madison M. Ahmad, Shannon D. Manning, Brian D. Gulbransen, Kevin R. Theis, Donald M. Kuhn

**Affiliations:** 1 Research and Development Service, John D. Dingell VA Medical Center, Detroit, Michigan USA; 2 Department of Psychiatry and Behavioral Neurosciences, Wayne State University School of Medicine, Detroit, Michigan USA; 3 Department of Biochemistry, Microbiology and Immunology, Wayne State University School of Medicine, Detroit, Michigan USA; 4 Department of Microbiology & Molecular Genetics, Michigan State University, East Lansing, Michigan USA; 5 Department of Physiology and Neuroscience Program, Michigan State University, East Lansing, Michigan USA; 6 Perinatal Research Initiative in Maternal, Perinatal and Child Health, Wayne State University School of Medicine, Detroit, Michigan USA

**Keywords:** Microbiome, Risk factors

## Abstract

Gulf War Illness (GWI) is a chronic health condition that appeared in Veterans after returning home from the Gulf War. The primary symptoms linked to deployment are posttraumatic stress disorder, mood disorders, GI problems and chronic fatigue. At first glance, these symptoms are difficult to ascribe to a single pathological mechanism. However, it is now clear that each symptom can be linked individually to alterations in the gut microbiome. The primary objective of the present study was to determine if gut microbiome dysbiosis was evident in a mouse model of GWl. Because the majority of Gulf War Veterans are overweight, a second objective was to determine if a high fat diet (HF) would alter GWI outcomes. We found that the taxonomic structure of the gut microbiome was significantly altered in the GWI model and after HF exposure. Their combined effects were significantly different from either treatment alone. Most treatment-induced changes occurred at the level of phylum in Firmicutes and Bacteroidetes. If mice fed HF were returned to a normal diet, the gut microbiome recovered toward normal levels in both controls and GWI agent-treated mice. These results add support to the hypotheses that dysbiosis in the gut microbiome plays a role in GWI and that life-style risk factors such as an unhealthy diet can accentuate the effects of GWI by impacting the gut microbiome. The reversibility of the effect of HF on the gut microbiome suggests new avenues for treating GWI through dietary intervention.

## Introduction

Soon after the end of hostilities in the Gulf War (August 1990–April 1991), a series of health issues began emerging in Gulf War Veterans and have persisted to the present day. The health issues reported are a perplexing and complex constellation of symptoms now known as Gulf War Illness (GWI). Over the past two decades, the Institute of Medicine has completed a series of studies on GWI and Health and the most recent review concluded that “Evidence is sufficient to conclude that a causal relationship exists between being deployed to the Gulf War and a health outcome” (p. 3^1^). When considering all symptoms that have been reported to be part of GWI, posttraumatic stress disorder was the only condition judged to have sufficient evidence of a causal relationship. The other symptoms for which evidence was sufficient to establish an association with deployment were mood disorders (anxiety, depression), GI symptoms (irritable bowel syndrome [IBS], dyspepsia) and chronic fatigue syndrome^1^. These disparate outcomes make it difficult to attribute GWI to a single mechanism until consideration is given to the gut microbiome.

The GI system of humans and most other mammals is inhabited by a very large number of bacteria, viruses, fungi and archaea. Collectively, these microorganisms make up the gut microbiome. It has been estimated that the gut contains 100 trillion cells and these cells express >150-fold more unique genes than the human genome^2^. The commensal members of the gut microbiome support human health but disruption in it has been implicated in a large number of clinical and physiological disorders [see^3–5^ for reviews]. Several conditions linked to enteric dysbiosis are reminiscent of GWI. First, IBS^6,7^ and dyspepsia^8^ are emerging as prototypical forms of gut dysbiosis. Second, the CNS symptoms associated with GWI (general anxiety, PTSD and depression) are frequently co-morbid with IBS and other inflammatory conditions of the bowel^9,10^. Third, chronic fatigue/fibromyalgia has also been linked to altered microbiome composition^11,12^. Therefore, the three main symptom clusters of GWI can be linked individually to gut dysbiosis, suggesting the possibility that a disrupted microbiome underlies all three. Indeed, a very small number of recent studies has confirmed that the gut microbiome is altered in Gulf War Veterans^13^ and in animal models of GWI^14–16^.

It remains perplexing that the symptoms of GWI are so chronic. In this preliminary report, we hypothesize that life-style risk factors, and specifically an unhealthy diet, could contribute to the persistence of GWI symptoms. It is known that Gulf War Veterans are often overweight or obese, both of which contribute to chronic health conditions^17,18^. Moreover, it is well known that a fat-laden diet causes dysbiosis within the human gut microbiome^19,20^, alters GI transit^21^ and can contribute to chronic low-grade gut inflammation (see^22^ for review). Animal studies have reported that energy dense^23^ and fat- or sugar-enriched diets^24^ not only cause significant alterations in the gut microbiome and fat accumulation but can also lead to changes in memory, brain inflammation and gut-brain communication. Germ-free mice colonized by fecal transfer from obese mice^25^ or obese humans^26^ develop significant increases in body fat, showing the importance of the microbiome in obesity. In this study, mice were exposed to a GWI model (pyridostigmine bromide (PB) and permethrin (PER)) and then fed either a normal diet (ND) or high fat diet (HF) to mimic conditions in Veterans with GWI. The results confirm that the gut microbiome is altered in an animal model of GWI and reveal that a HF further alters the dysbiotic gut microbiome in this model.

## Materials and Methods

### Animal model of GWI

An established mouse model of GWI, as effectively employed by Crawford and colleagues^27–30^, was used in the present studies. This model has been extensively validated^31^ and has been deemed a GWI-relevant animal model in The Gulf War Illness Landscape (https://cdmrp.army.mil/gwirp/pdfs/GWIRP_Landscape.pdf) published by the DoD GWI Research Program. Male C57BL6/J mice (8 weeks of age) were purchased from Envigo (Indianapolis, I.N.) and housed individually in a room with constant temperature and humidity and with alternating 12 hr periods of light and darkness. All mice used in these studies were from the same cohort and assignment to treatment groups was random. Half of the mice were injected with 50 μl of GWI agents in final doses of 0.7 mg/kg of pyridostigmine bromide (PB) and 200 mg/kg of permethrin (PER) solubilized in 100% dimethyl sulfoxide (DMSO). Drug solutions were further diluted with sterile physiological saline to a final DMSO concentration of 3% just prior to intraperitoneal injection. The other half served as controls and received intraperitoneal injections of 3% DMSO in sterile physiological saline. Injections were administered once daily for 10 days. Several studies consistently show that in rodents, exposure to PER + PB results in neurobehavioral alterations (i.e. anxiety and mood impairment) that are similar to symptoms reported by Veterans with GWI^28^. Thus, anxiety and depression-like behaviors were tested as specified below. During treatment, all mice were given ad libitum access to water and normal rodent laboratory chow ((ND); D12450K with 10 kcal% from fat, Research Diets, New Brunswick, NJ). On the last day of treatment, the GWI and control groups were split into 3 same sized groups (N = 7–9 mice per group) and fed the following diet regimens: one group on a ND and two groups on a high fat diet ((HF); D12451 with 45% kcal from fat, Research Diets, New Brunswick, N.J.) known to induce obesity in mice^32,33^). After 3 weeks, one of the HF fed groups was switched back to a ND while the two other groups were continued on their original HF or ND for an additional 3 weeks. Hereafter, the treatment/diet groups are referred to as Con-ND-ND, Con-HF-HF and Con-HF-ND for controls and GWI-ND-ND, GWI-HF-HF and GWI-HF-ND for PER + PB treated mice. To validate the GWI model at the specific post-treatment time of 6 weeks that mice were exposed to diets, the Con-ND-ND and GWI-ND-ND groups were evaluated for depression- (splash test) and anxiety- (elevated plus maze) like behaviors prior to sacrifice. These are two of the core components of mood disorders present in individuals with GWI^1^. The splash test was performed according to our previously reported work^34^. Briefly, this test involves spraying a 10% sucrose solution onto the dorsal coat of the mouse in its home cage. This mildly sticky solution induces self-grooming, and the time the mouse spends grooming is considered a direct measure of self-motivated care. The elevated plus maze was also performed according to our previous reports^35^. In this test, the time spent in both the open and closed arms of the maze was recorded for each mouse in 5 min sessions using a motion-sensitive digital video camera and EZ Video freeware Software (Ezvid, Inc, Los Angeles, CA; https://www.ezvid.com/ezvid_for_windows). Mice were sacrificed by decapitation and the contents of the caecum were harvested and frozen at −80°C. Stressors such as noise and handling by multiple persons were avoided and mice were monitored daily for signs of distress or injury until the experimental endpoint. The Institutional Care and Use Committee of Wayne State University approved the animal care and experimental procedures (IACUC 17-08-0307). All procedures were also in compliance with the *NIH Guide for the Care and Use of Laboratory Animals* and were conducted in compliance with ARRIVE guidelines and under IACUC-approved protocols.

### Microbiome analysis

DNA was extracted from caecum contents (~200 mg wet weight) using QIAamp PowerFecal DNA kits and sample DNA concentrations were determined using a Qubit 4 Fluorometer (range 70–100 ng/µl). Samples were sequenced in duplicate on an Illumina MiSeq system using a 2 × 250 cycle V2 kit with Illumina reagents and Illumina sequencing procedures detailed by Kozich and colleagues^36^. The 16S rRNA gene primers targeted the V4 region of the gene (forward primer: 5′-GTGCCAGCMGCCGCGGTAA-3′; reverse primer: 5′-GGACTACHVGGGTWTCTAAT-3′). The 16S rRNA gene sequences from the paired fastq files were trimmed, screened and aligned using mothur^37^, in accordance with the MiSeq SOP established by Schloss and colleagues (https://www.mothur.org/wiki/MiSeq_SOP). After de-multiplexing and quality control (e.g., truncating reads with >2 adjacent low quality base calls; discarding reads containing any ambiguous base calls in surviving sequences), sequences were binned into operational taxonomic units (OTUs) based on percent sequence identity (97%). The OTUs were taxonomically classified in mothur, and the bacterial community data were thereafter visualized and statistically analyzed using PAST software (v3.20^38^). Microbiome diversity was characterized in terms of α-diversity using the Chao1 (i.e. community richness) and Shannon and Simpson (1-D) (i.e. community heterogeneity) indices. The number of sequences and Good’s coverage values were analyzed using one-way ANOVA. β-diversity was assessed using the Jaccard (i.e. shared composition) and Bray-Curtis (i.e. shared structure) indices based on relative abundance data. High-dimensional class comparisons were carried out with linear discriminant analysis effect size (LEfSe) in an on-line interface^39^ using default parameters except that the minimum LDA score was set to 3.6. Heat maps were generated using MetaboAnalyst 4.0^40^.

### Data analysis and statistics

Data from splash test was analyzed with an unpaired student’s t test using GraphPad Prism (v6.07) for Windows (GraphPad Software, La Jolla, CA, USA, www.graphpad.com). Time spent in each set of arms of the elevated plus maze was analyzed by two-way ANOVA and subsequent Sidak’s multiple comparison tests. Food and body weight data were analyzed with two-way ANOVA followed by Tukey’s *post hoc* tests using Prism The indices for α-diversity were obtained using PAST software (v3.20; free software for scientific data analysis, Oyvind Hammer, Natural History Museum, University of Oslo, Norway; https://folk.uio.no/ohammer/past/). The results were analyzed statistically with a one-way ANOVA and subsequent Tukey’s *post hoc* comparisons, using Prism. The indices for β-diversity were also calculated, and statistical analyses were carried out, using PAST software as well. The results were analyzed using a two-way NPMANOVA, and *post hoc* comparisons were made using one-way NPMANOVAs. Taxonomic distributions at the phylum level (treatment X phylum) and lower taxonomic levels (treatment X time) were analyzed with a two-way ANOVA followed by *post hoc* comparisons using Tukey’s tests in GraphPad Prism.

## Results

### Effects of HF on food intake and body weight in a model of GWI

**Figure 1:**
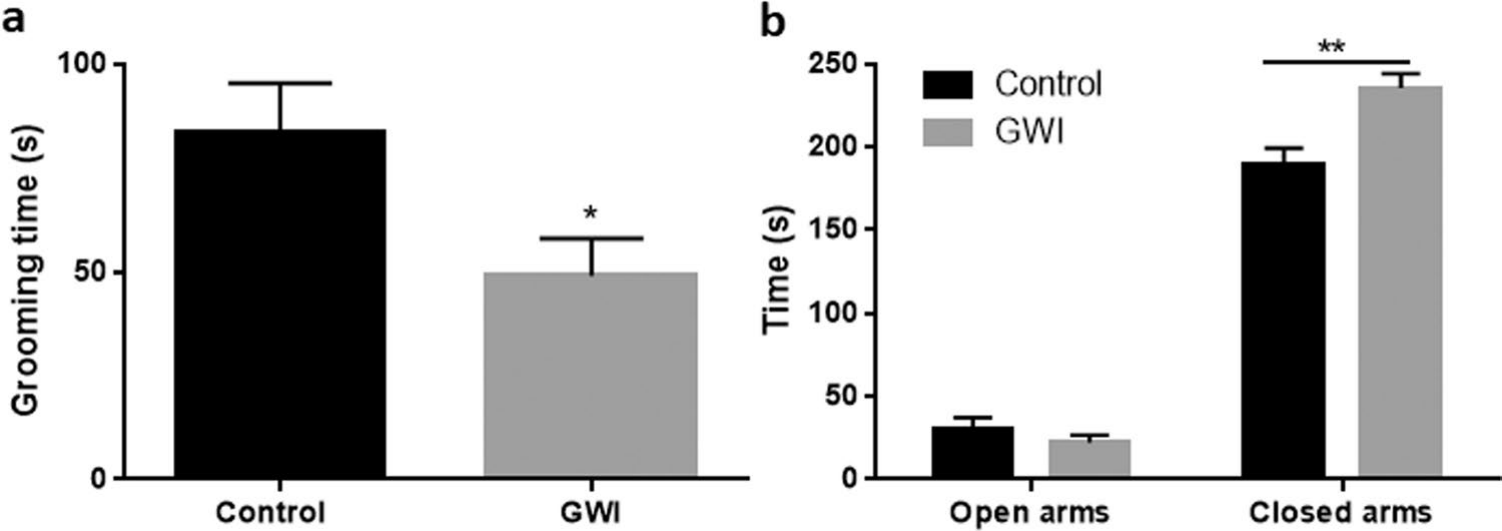
Effects of treatment with Gulf War agents PER + PB on the splash test (**A**) and the elevated plus maze (**B**). Behaviors were evaluated 6 weeks after administration of the agents to corroborate that the GWI model induced some of the outcomes reported for this condition. Results are mean ± SEM, N = 5–6. Symbols represent significance levels for the indicated comparisons as p < *0.05, **0.01.

Figure 1 shows that the GWI model used in this study recapitulates some of the key features of the condition, such as mood alterations. Mice treated with PER + PB showed decreased self-motivated care reflected as a shorter grooming time in the splash test compared to controls (p < 0.05; Fig. 1A). This is associated with a depression-like phenotype in rodents. Two-way ANOVA analysis of anxiety-like behaviors tested with the elevated plus maze test revealed a main effect of treatment (F_1,20_ = 6.64, p < 0.05), time in each set of arms (F_1,20_ = 633.2, p < 0.0001) and these two factors interaction (F_1,20_ = 13.51, p < 0.01). The time animals treated with PER + PB spent in the closed arms of the maze was significantly longer compared to controls (p < 0.01, *post hoc* Sidak’s test), whereas no differences were found in the time spent in the open arms (Fig. 1B). These results are indicative of anxiety-like phenotype in the mice treated with PER + PB.

**Figure 2:**
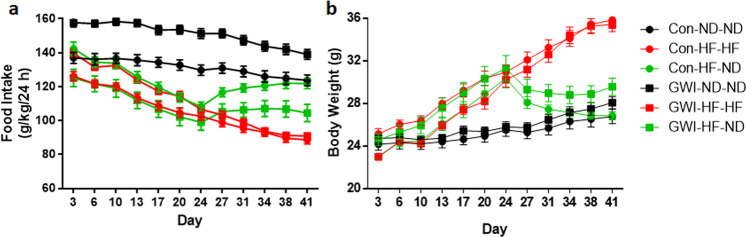
Effect of diet on food intake (**A**) and body weight (**B**). Mice were treated with GWI agents or Con (control) and then fed a normal (ND) or high fat (HF) diet for 3 weeks. Thereafter, half of the mice on the HF diet (Con and GWI) were switched to ND (HF-ND) for an additional 3 weeks. Remaining GWI and Con mice were fed ND or HF diet throughout (ND-ND or HF-HF). Food intake measures were calculated based on food consumption (g), mouse body weight (kg) for a 24 h period and reported as g/kg/24 h. Results are mean ± SEM, N = 7–9.

Figure 2 A shows food intake measures for all groups and analysis by two-way ANOVA revealed significant main effects of time (F_11,484_ = 72.71, p < 0.0001), treatment (F_5,44_ = 65.82, p < 0.0001) and their interaction (F_55,484_ = 5.57, p < 0.0001). The GWI agent-treated group displayed a significantly higher food intake of the ND compared to controls fed equally (*post hoc* Tukey’s test; p < 0.0001). The consumption of HF impacted the food intake as Con-HF-HF mice had a lower intake compared to Con-ND-ND mice (*post hoc* Tukey’s test; p < 0.0001) and to Con-HF-ND (*post hoc* Tukey’s test; p < 0.0001). Con-ND-ND mice did not differ from Con-HF-ND. In mice treated with GWI agents, both groups fed with HF showed a decreased intake compared to mice fed with ND (*post hoc* Tukey’s tests for both GWI-HF-HF and GWI-HF-ND vs GWI-ND-ND; p < 0.0001). No differences were detected when comparing the GWI-HF-HF group to the GWI-HF-ND mice. While HF was associated with a lower food intake, body weight followed the opposite trend (Fig. 2B). Both treatment groups fed the HF (Con-HF-HF and GWI-HF-HF) gained on average 10 g over the 6 week test period, whereas both groups fed the ND (Con-ND-ND and GWI-ND-ND) gained ~3 g. Body weight was not altered by treatment with GWI agents as Con-ND-ND was not different from GWI-ND-ND, and Con-HF-HF was not different from GWI-HF-HF mice. When mice initially fed a HF diet were switched to the ND for 3 weeks, both groups lost significant amounts of weight (~6–7 g). However, the Con-HF-ND group achieved a significant reduction in body weight sooner after the diet switch (*post hoc* Tukey’s test; p < 0.001 at day 27) than the GWI-HF-ND group (*post hoc* Tukey’s test; p < 0.001 at day 31), and the GWI agent-treated mice ultimately lost less weight than controls (*post hoc* Tukey’s test; p < 0.05 at day 41). The main effects of time (F_11,528_ = 64.8, p < 0.001) and treatment (F_5,528_ = 115.9, p < 0.001) as well as their interaction (F_55,528_ = 7.3, p < 0.001) were significant (2-way ANOVA). These data establish that the HF led to significant gains in body weight that were of the same magnitude in controls and mice treated with GWI agents. Both groups lost significant weight when switched back to ND, although weight loss was more pronounced among controls.

### Effects of treatment with GWI agents and HF on the gut microbiome at the OTU level

The number of sequences obtained were as follows: 117,212 ± 7,509 for Con-ND-ND, 103,432 ± 17,384 for Con- HF-HF, 128,772 ± 9,319 for Con-HF-ND, 100,369 ± 10,433 for GWI-ND-ND, 111,781 ± 32,363 for GWI-HF-HF, and 128,371 ± 32,694 for GWI-HF-ND. There were no statistically significant differences among these groups with respect to sequence numbers. Good’s coverage values ± SD were the following: 99.63 ± 0.043 for Con-ND-ND, 99.7 ± 0.056 for Con-HF-HF, 99.67 ± 0.025 for Con-HF-ND, 99.63 ± 0.025 for GWI-ND-ND, 99.71 ± 0.06 for GWI-HF-HF, and 99.69 ± 0.079 for GWI-HF-ND.

**Figure 3:**
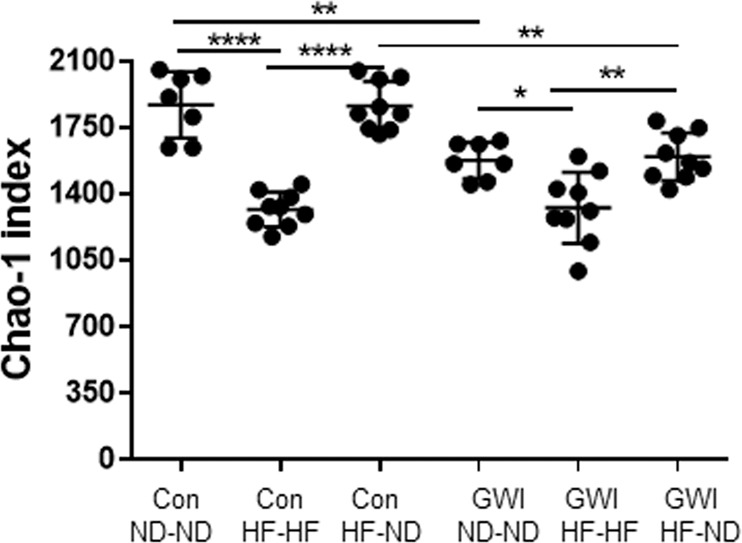
Effects of GWI ± HF on α-diversity. Data are presented as Chao-1 ± SEM, N = 8-9. Con = control; GWI = PER + PB; ND = normal diet; HF = high fat diet. Symbols represent significance levels for the indicated *post hoc* comparisons as p < *0.05, **0.01, ****0.0001.

Figure 3 presents an analysis of α-diversity using the Chao-1 index as a measure of gut microbiome richness. The main effect of treatment (F_5,44_ = 26.1, p < 0.0001) was significant. *Post hoc* comparisons indicated that treatment with GWI agents significantly reduced microbiome richness compared to controls (Tukey’s test, p < 0.05), and that HF led to significantly decreased richness in both control (Tukey’s test, p < 0.001) and GWI agent-treated groups (Tukey’s test, p < 0.05). Notably, when mice were shifted from HF to ND, α-diversity recovered to the levels of the appropriate treatment control and differed significantly from the respective HF-HF group (Tukey’s test, p < 0.001 for controls and p < 0.01 for GWI).

**Figure 4:**
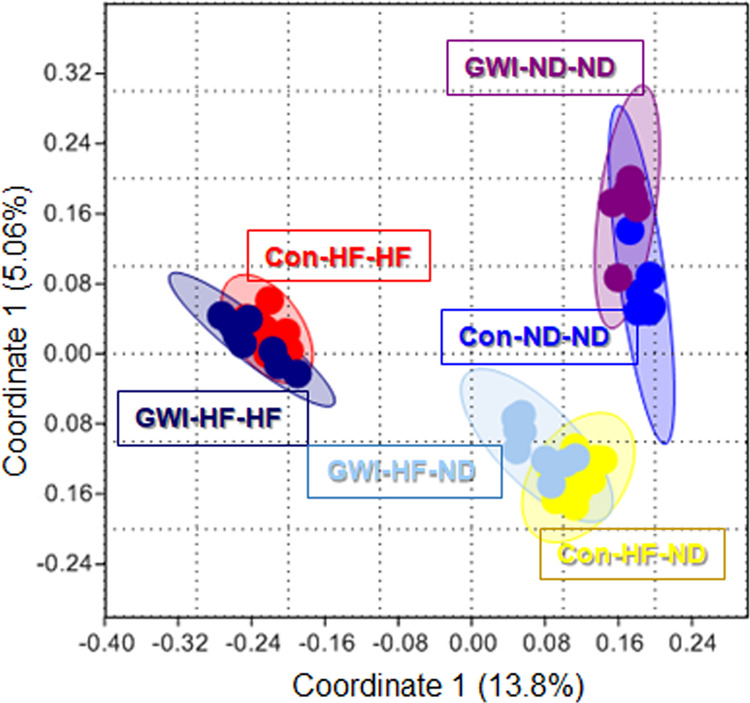
Effects of treatments on β-diversity. PCoA showing differences in the similarities of the gut microbiome profiles of the study groups using the Jaccard index. Con = control; GWI = PER + PB; ND = normal diet; HF = high fat diet.

Results of α-diversity analyses based on the Simpson (1-D) index indicated that, while the heterogeneity of the gut microbiome did not differ between GWI agent-treated mice and controls, gut microbiome heterogeneity was consistently highest in HF mice whereas there were no consistent effects of treatment on gut microbiome heterogeneity using the Shannon index (Supplementary Fig. S1). With respect to β-diversity, analyses based on the Jaccard index, which reflects shared microbiome membership (i.e. community composition) results showed that the OTU profiles of samples clustered together tightly according to the diet regimen, and that within diet regimen groups, samples also clustered by treatment (Fig. 4). Two-way NPMANOVA analyses revealed that the main effects of treatment (p < 0.01) and diet (p < 0.0001), as well as their interaction (p < 0.02), were significant. All *post hoc* comparisons among groups were statistically significant (Supplementary Table S1). It is interesting that mice in the control and GWI agent-treated groups exposed to the HF-ND regimen clustered near the ND-ND groups on the PCoA plot, suggesting rapid recovery of the gut microbiome following a return to a ND, as was also seen above for α-diversity. Results for β-diversity using the Bray-Curtis index, which reflects overall microbiome structure (i.e. not just membership), were similar to those for the Jaccard index (Supplementary Fig. S2).

**Figure 5:**
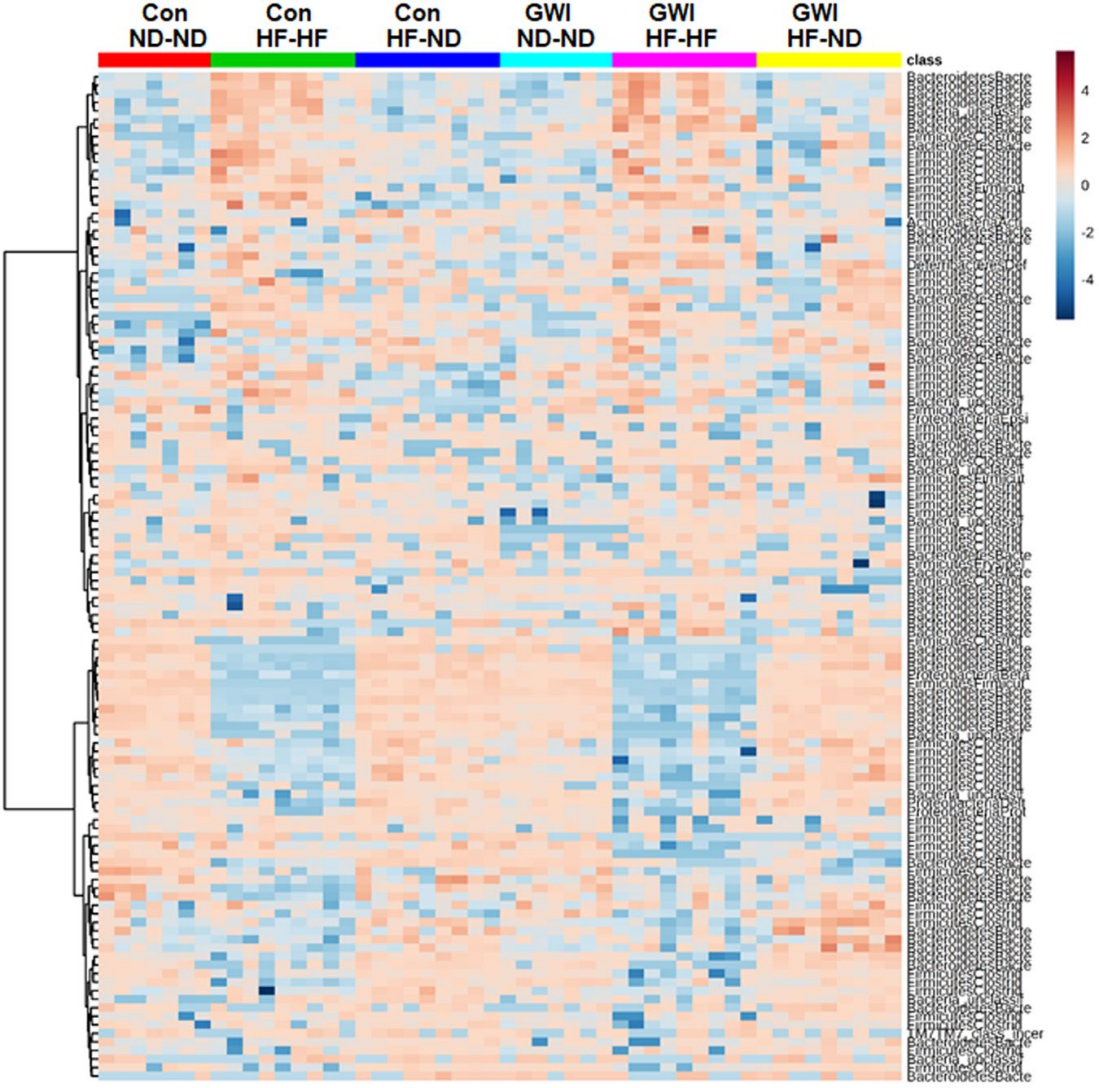
Heat map illustrating patterns in OTU relative abundance among the treatment groups. All subjects in each group are arrayed in columns and bacterial taxonomies are indicated in rows. Con = control; GWI = PER + PB; ND = normal diet; HF = HF diet. Clustering along the y-axis was done using the Ward algorithm.

The taxonomic identities of prominent OTUs (≥1.5% average relative abundance among all subjects) varied among treatment groups. These results are presented in the heat map in Fig. 5. It can be seen that the GWI agent-treated and control groups displayed similar patterns of OTU expression according to diet. The most prominent differences in these groups were decreases in Bacteroidetes (see the clusters near the bottom of Fig. 5) and increases in Firmicutes (clusters near the top) in the Con-HF-HF and GWI-HF-HF groups. Furthermore, within each diet group, differences in OTU relative abundances were evident for the GWI agent-treated versus controls. As reported above for community α and β diversity, as mice in the GWI agent-treated and control groups transitioned from HF to the ND, patterns of OTU relative abundance appeared to “recover” toward the pattern shown in the groups fed ND throughout this experiment (i.e., ND-ND groups).

**Figure 6:**
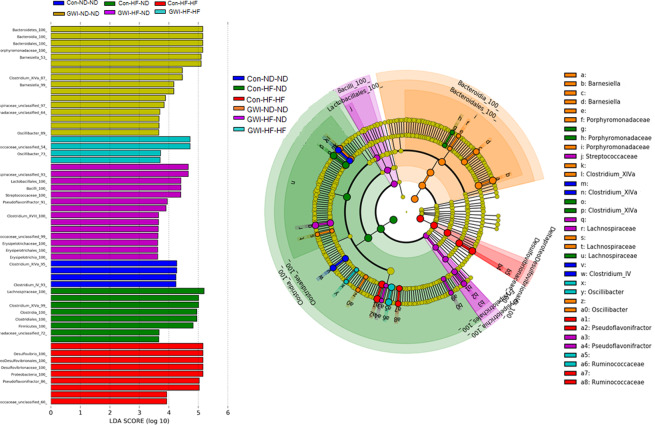
Bacterial taxa that were differentially abundant across treatments. LEfSe was carried out using the Galaxy Project and the results are displayed in the bar charts (**A**) and the associated cladogram (**B**). Taxa showing different abundance values in each treatment group (according to LEfSe) are shown in the cladogram highlighted by small circles and by shading. All groups are statistically significant compared to each other (LDA > 3.6). Con = control; GWI = PER + PB; ND = normal diet; HF = high fat diet.

Figure 6 presents results from linear discriminant analysis effect size (LEfSe) analysis and highlights the effect sizes of the treatments and diets on affected taxa. LEfSe compares each group to all others simultaneously and generates bar plots that include taxa that are distinctly relatively abundant in each specific treatment and diet group. Segata *et al*.^39^ propose LEfSe as a means for biomarker discovery by finding OTUs that consistently explain the differences between two or more types of microbial communities. Two main outcomes from this analysis are apparent. First, the groups treated with GWI agents are demarcated by more taxonomic biomarkers than controls for each diet condition. Second, most treatment groups were distinguished by taxa in the order Clostridiales within the phylum Firmicutes (i.e., Con-ND-ND, Con-HF-ND and GWI-HF-ND). However, the GWI-ND-ND group was represented primarily by taxa in the order Bacteroidales within the phylum Bacteroidetes, the Con-HF-HF group was singularly characterized by taxa within the order Desulfovibrioales, and the GWI-HF-HF group was represented by taxa within the orders Lactobacillales and Erysipelotrichales. The HF diet shifted the predominant taxa for the GWI-ND-ND group from Bacteroidetes to Firmicutes. All of the control groups regardless of diet were distinguished by taxa within Firmicutes and the relatively most abundant taxa in the group fed a ND were in the *Clostridium XIVa and IV* clusters. Controls fed the HF diet were characterized by taxa within the genera *Desulfovibrio* and *Pseudoflavonifractor* and the control group shifted to a ND from the HF diet was distinguished by Porphyromonadaceae and Lachnospiraceae. Treatment- and diet-induced biomarkers were observed down to the level of family or genus as shown in the cladogram (Fig. 6).

### Effects of treatment with GWI agents and HF on the gut microbiome at the phylotype level

**Figure 7:**
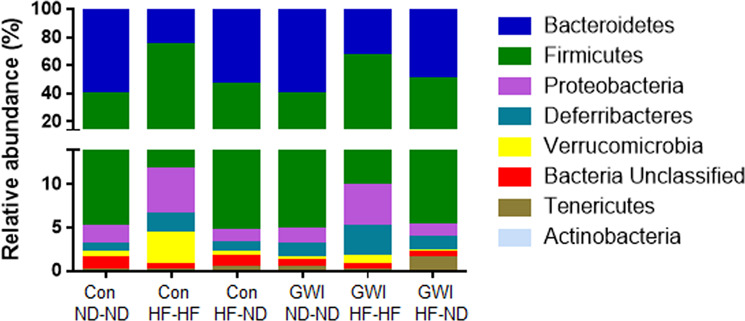
Percent relative abundances of phyla in treatment and diet groups. Stacked columns for the 8 most prominent phyla are included. Con = control; GWI = PER + PB; ND = normal diet; HF = high fat diet.

Figure 7 illustrates treatment effects at the phylotype level. Treatment and diet effects on specific bacterial phyla are presented as percent relative abundance. The main effect of phylum was significant (F_7,352_ = 2616, p < 0.0001) but the treatment main effect was not. The phylum X treatment interaction was also significant (F_35,352_ = 50.6, p < 0.0001) by two–way ANOVA. *Post hoc* comparisons revealed that virtually all treatment groups differed significantly from one another (p values ranging from 0.05 to 0.0001). The observed changes occurred only within the prominent phyla Firmicutes and Bacteroidetes (Fig. 7). The only groups that did not differ were Con-ND-ND vs GWI-ND-ND within Firmicutes and Con-ND-ND vs GWI-ND-ND within Bacteroidetes. The results of all pairwise statistical tests for % relative abundance of Firmicutes and Bacteroidetes among treatment groups are presented in Supplementary Table S2.

**Figure 8:**
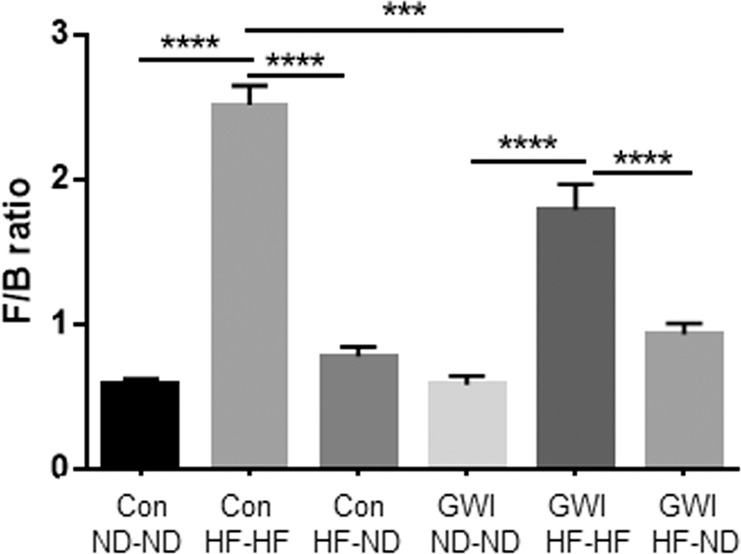
Firmicutes to Bacteroidetes (F/B) ratio in treatment and diet groups. Results are presented as means + SEM for each treatment and diet. Symbols represent significance levels for the indicated *post hoc* comparisons as p < : ***0.001, ****0.0001. Con = control; GWI = PER + PB; ND = normal diet; HF = high fat diet.

Because the observed differences in % relative abundance occurred within the Firmicutes and Bacteroidetes phyla, and in light of the findings that the ratio of Firmicutes/Bacteroidetes (F/B) is higher in obese and overweight humans than in lean controls^41^, we calculated this ratio for all treatment groups and the results are presented in Fig. 8. The main effect of treatment was significant (F_5,44_ = 50.8, p < 0.0001). Specifically, the HF diet caused significant increases in the F/B ratio for controls and GWI treated mice (Tukey’s test, p < 0.0001 for both). The increase in the F/B ratio was significantly greater in the control mice than the GWI agent-treated mice (Tukey’s test, p < 0.001). When groups fed the HF were shifted to the ND, the F/B ratio decreased to levels observed in the respective ND-ND controls (Tukey’s test, p < 0.0001 for both).

### Effects of treatment with GWI agents and HF on taxa below the level of phylum

**Figure 9:**
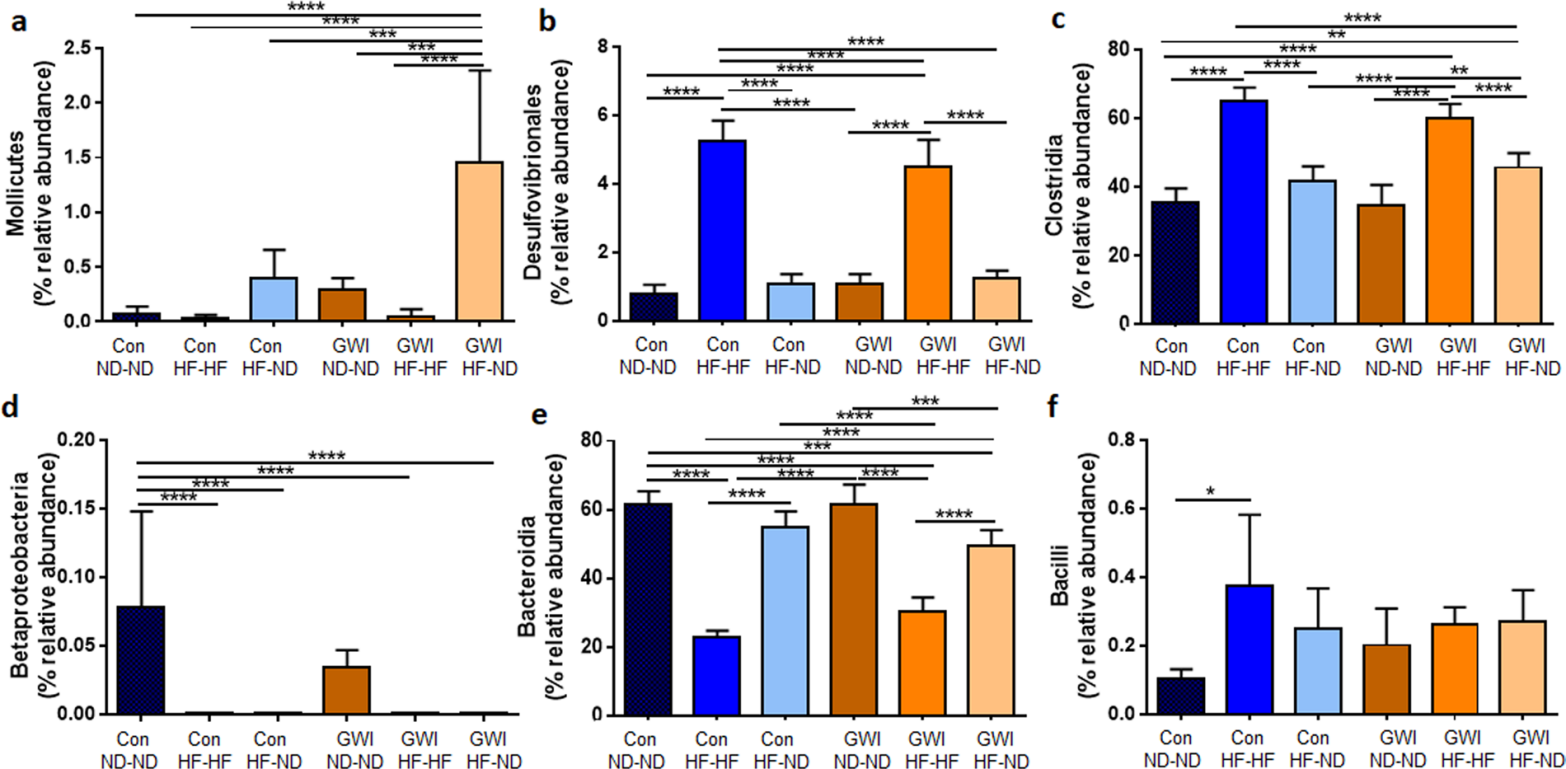
Relative abundance of taxa below the level of phylum in treatment and diet groups. Results are presented as % relative abundance for each taxon. Con = control; GWI = PER + PB; ND = normal diet; HF = high fat diet. Symbols represent significance levels for the indicated *post hoc* comparisons as p < : *0.05, **0.01, ***0.001, ****0.0001.

The effects of treatments and diets on taxa below the level of phylum were also probed in view of the likelihood that changes at the highest taxonomic level may have not reached statistical significance because of increases and decreases of equal magnitude within phyla in percent relative abundances of bacteria at lower taxonomic levels. Figure 9 shows these results and indicates that effects at the taxonomic levels of class and order vary in a complex manner that is dependent on the combined influence of treatment and diet. The main effect of treatment in each panel of Fig. 9 was significant by one-way ANOVA with p values ranging from 0.035 (for Bacilli) to 0.0001 (for all remaining taxa). In general, the effects of the HF on bacterial taxa were more prevalent than those of GWI-agents treatment. The Con-ND-ND group did not differ from the GWI-ND-ND group, whereas both control and GWI agent-treatment groups fed ND-ND were significantly different from the respective HF-HF groups for most taxa. The complexity of the changes are most evident for Desulfovibrionales and Clostridia, where the relative abundances of these taxa were increased in HF-HF groups compared to ND-ND groups, and in Betaproteobacteria and Bacteroidia, which were both greatly decreased in abundance in the HF-HF groups. Two additional unique changes can be seen in Fig. 9A where the abundance of Mollicutes in GWI-HF-ND group was significantly increased compared to the other groups, and in Fig. 9D where the abundance of Betaproteobacteria was significantly decreased for most groups compared to the Con-ND-ND group.

Each of the OTUs from the LEfSe analysis (Fig. 6) was subjected to analysis using the Basic Local Alignment Search Tool (BLAST) in an attempt to identify taxa that were differentially abundant among treatments at the species level (i.e. the consensus sequence of the OTU had >99% sequence identity with the sequence of a bacterial species within the BLAST taxonomy database). The results presented in Table 1 show that all groups except Con-HF-ND were represented by specific bacterial species. The Con-ND-ND group was characterized by *Muribaculum intestinale* whereas *Fusimonas intestini* was characteristic of the GWI-ND-ND group. The Con-HF-HF group was represented by *Flintibacter butyricus* and *Bacteroides intestinalis* and the corresponding GWI-HF-HF group was demarcated by *Bacteroides vulgatus, Mucispirillum schaedleri* and *Parabacteroides goldstenii*. Finally, the biomarkers *Paramuribactum intestinale, Duncaniella muris* and *Bacteroides acidifaciens* emerged in the GWI-HF-ND group.

**Table 1:** Bacterial species identified by BLAST analysis.

OTU #	Phylum	Bacteria sp	Identity (%)	Group
OTU0088	Bacteroidetes	*Muribaculum intestinale*	100	Con-ND-ND
OTU0007	Firmicutes	*Flintibacter butyricus*	99.6	Con-HF-HF
OTU0075	Bacteroidetes	*Bacteroides intestinalis*	99.6	Con-HF-HF
OTU0047	Firmicutes	*Fusimonas intestini*	99.6	GWI-ND-ND
OTU0022	Bacteroidetes	*Paramuribaculum intestinale*	100	GWI-HF-ND
OTU0066	Bacteroidetes	*Duncaniella muris*	100	GWI-HF-ND
OTU0011	Bacteroidetes	*Bacteroides acidifaciens*	100	GWI-HF-ND
OTU0019	Bacteroidetes	*Bacteroides vulgatus*	100	GWI-HF-HF
OTU0013	Deferribacteres	*Mucispirillum schaedleri*	100	GWI-HF-HF
OTU0069	Bacteroidetes	*Parabacteroides goldstenii*	100	GWI-HF-HF

## Discussion

The goal of the present study was to determine if a HF would interact with PER and PB to further alter the gut microbiome in a mouse model of GWI. The rationale for this pilot study was the fact that a majority of Gulf War Veterans are overweight or obese^17,18^, and that fat-laden diets can lead to changes in memory, GI and brain inflammation and gut-brain communication^19,20,23,24^. In this regard, it was important to rule out that an increased caloric consumption of the HF diet rather than its fat component itself was responsible for the observed effects. Thus, the energy density for the groups fed with HF versus ND was calculated. According to manufacturer’s specifications, the energy density for the ND is 3.8 Kcal/g, whereas for the HF it is 4.7 Kcal/g. Using an average of the intake of each diet group over the entire experiment, the caloric densities were surprisingly higher for the ND groups (490.96 Kcal for Con-ND-ND and 530.1 Kcal for GWI-ND-ND) than for the HF groups (443.1 Kcal for Con-HF-HF and 444.4 Kcal for GWI-HF-HF). This is evidence that the number of calories was not the causative factor for the effects we reported. The experimental results established that PER and PB caused a significant dysbiosis, as did exposure to a HF, and their combined effects led to an altered gut microbiome that was significantly different from the effect of either treatment alone. These results are even more impactful when considering the relatively short-term period over which mice were fed the HF (i.e., 3 or 6 weeks). Consumption of the HF for only three weeks caused significant increases in body weight in groups treated with PER + PB or controls compared to mice maintained on a ND. Two additional observations link these effects to alterations in the gut microbiome as a mediating factor. First, the Gulf War agents PER and PB did not alter water intake or the amount of food consumed on either diet. Second, when mice in both treatment groups were shifted from the HF to a ND for three additional weeks, mice treated with PER + PB lost significantly less weight than controls.

PER + PB and diet each caused significant alterations in the taxonomic makeup of the gut microbiome. The predominant changes in OTU structure occurred within the Firmicutes and Bacteroidetes phyla. This pattern was expected in light of the fact that the mouse gut microbiome is dominated by these two phyla^42^. Treatment with PER + PB caused a complex set of alterations in α-diversity. In both GWI agent-treated and control mice, those fed HF diets throughout the duration of the experiment exhibited gut microbiomes with reduced richness. Nevertheless, the gut microbiomes of all mice in the experiment remained OTU-rich, with Chao1 index values exceeding 1000. This high degree of OTU-richness resulted in high values for microbiome heterogeneity as well, with Simpson (1-D) and Shannon index values exceeding 0.93 and 4.0, respectively. The heterogeneity of gut microbiomes from HF-HF mice exceeded that of ND-ND mice in both GWI agent-treated and control groups based on the Simpson index, but not the Shannon index. These data suggest that although HF led to a reduction in the OTU-richness of the gut microbiome, the OTUs that were present in the guts of HF-treated mice were more evenly distributed in their relative abundances than were the OTUs in the gut microbiomes of ND mice.

Treatment with PER and PB and the HF each led to significant alterations in the complexity of the gut microbiome. The OTUs for the different diet conditions clustered together tightly and apart from the other groups. Mice fed the HF throughout (HF-HF) were most distant from mice fed a ND throughout (ND-ND) on the PCoA plot. Interestingly, when GWI and control mice were shifted from the HF to a ND (HF-ND), both groups clustered nearest to their respective ND-ND groups, suggesting a partial recovery in β-diversity after the dietary shift. Nevertheless, within each diet condition cluster, the GWI agent-treated mice differed significantly from controls. These results emphasize the fact that a life-style risk factor such as a HF can accentuate the effects of PER and PB on community diversity and establish the reversible nature of this effect after return to a ND.

LEfSe analysis identified numerous bacterial taxa that were differentially abundant among treatment groups and these taxonomic “biomarkers” varied substantially between the GWI agent-treated mice and those exposed to dietary shifts. The gut microbiome in mice fed the ND throughout were dominated by *Clostridium XIV* whereas the mice treated with PER + PB were dominated by *Barnesiella* and Porphyromonadaceae. The HF resulted in a large increase in the predominant taxa for both GWI and control mice. For instance, the GWI agent-treated mice on a HF were most defined by *Enterococcus, Clostridium*, Porphyromonadaceae, *Oscillibacter* and Proteobacteria whereas controls were dominated by *Clostridium XIV*, Ruminococcaceae, Erysipelotochaceae, *Barnsiella*, Lachnospiraceae and Actinobifidobacteriales. As seen above in treatment-induced alterations in community diversity, the shift from a HF to a ND led to a reduction in the number of defining taxa for both GWI agent-treated mice and their controls. Many of the differentially abundant taxa that emerged in the HF-HF groups (by comparison to the ND-ND groups) were not evident in the HF-ND mice for both GWI and control groups although the number of remaining taxa was greater than that seen in the ND-ND groups.

The individual OTU’s that were identified in the LEfSe analysis were compared to 16S rRNA gene sequence data in the NCBI data base using BLAST in an attempt to identify bacterial species that were markers for the present treatment groups. A total of 10 species were matched with 99.6 to 100% sequence identity with 3 species linked to the Con-ND-ND and Con-HF-HF groups and 7 linked to the GWI agent-treated groups in all dietary conditions. Of these, 7 species were from the Bacteroidetes phylum, 2 were from Firmicutes and 1 was from Deferribacteres. Some interesting parallels to GWI can be seen in the identified species. For instance, *Flintibacter butyricus*, which was a marker for the Con-HF-HF group is increased in mice fed bile acids and a dietary fat^43^. *Mucispirillum schaedleri* was relatively most abundant in the GWI-HF-HF group and is known to be expanded in the gut under inflammatory conditions accompanied by reactive oxygen/nitrogen stress^44^. The GWI-HF-ND group was characterized by *Bacteroides acidifaciens* and *Duncaniella muris*. *B. acidifaciens* can ameliorate metabolic disorders such as diabetes and obesity and is expanded in lean phenotypes of the atg7 knockout mouse^45^. When mice fed a HF supplemented with resistant starch, the starch caused significant improvements in the intestinal health of obese mice and was associated with expansion of *D. muris*^46^.

It is not yet possible to draw direct associations between a GWI model and HF to specific gut microbiome alterations. This can be attributed to several different factors. First, rodent models are probably limited in the extent to which they mimic the conditions to which Gulf War Veterans were exposed during their deployment. Second, GWI is a heterogeneous disorder making it difficult to link it to changes in specific taxa. For example, increases in Proteobacteria have been linked to gut inflammatory conditions^47^ including a preliminary study of GWI^13^. While our present results showed significant increases in Proteobacteria, in the Con-HF-HF group, this increase did not quite reach statistical significance in the GWI agent-treated groups. The present results did document a significant increase in the F/B ratio for groups fed the HF-HF diet (both controls and GWI) in agreement with data from humans with IBS^6^. A more recent meta-analysis suggests that at least IBS is characterized at the genus level by decreases in *Lactobacillus* and *Bifidobacterium* and increased levels of *Escherichia coli* and *Enterobacter* (both in the Proteobacteria phylum) without changes in Bacteroidetes and *Enterococcus*^6^. Both of these outcomes are not fully recapitulated in Veterans with GWI^13^ or in rodent models of this disorder^14–16^, including the results of the present study. Third, GWI is not IBS and likely encompasses a different set of pathological alterations such that some Veterans with GWI have GI disturbances while others do not^1,13^.

The present results stand in contrast to a recent study showing gut microbiome alterations in a mouse model of GWI^14^. Alhassan and colleagues demonstrated that mice treated with Gulf War agents plus corticosterone showed a significant increase in OTU richness and higher percent relative abundances for Firmicutes and Tenericutes over Bacteroidetes at the level of phylum. In contrast, we observed a reduction in OTU richness with GWI treatment in both the ND-ND and HF-ND groups and we did not observe increases in the relative abundance of Tenericutes in GWI agent-treated mice. These discrepancies may reflect differences in the Gulf War models used, the use of different survival times post-treatment, and the lack of a stress-only group in the Alhassan *et al*.^14^ study. Despite the differences in these two preclinical studies, the possibility that gut microbiome alterations may play a role in the symptoms of GWI is strengthened by the recent report of dysbiosis in Gulf War Veterans^13^.

The present study has several strengths. First, it adds support to the hypothesis that gut microbiome dysbiosis contributes to the symptoms of GWI. Second, it is the first characterization of the effect of a life-style risk factor–a diet rich in fat–on the alterations in the gut microbiome caused by PER + PB. Life-style risk factors that contribute to poor health could play important roles in extending the duration and severity of the symptoms of GWI and may help explain how the symptoms of GWI persist for so long after PER and PB levels have dropped below detection in Gulf War Veterans^1^. Third, we show that the interaction between treatment with GWI agents and diet is significant, such that their combined effects on the gut microbiome are greater than either treatment alone. Fourth, the present study shows that the enhancement of gut microbiome dysbiosis by a HF in a model of GWI is reversible and leaves open the possibility that dietary modifications or other non-invasive treatments that alter the gut microbiome (e.g., probiotics, antibiotics) may provide relief from the symptoms of this chronic multi-system disorder.

Our study has three primary limitations. First, it is a molecular microbiology study without experiments designed to link gut microbiome alterations in a GWI model to changes in GI (e.g., leakiness, inflammation). Second, this project had a single post-treatment survival time; future experiments should include exposure to a HF for longer periods of time (e.g., 3–6 months) to evaluate the impact on severity and chronicity of GWI symptoms. Third, it cannot yet be determined if the observed effects of the GWI agents are due to direct effects on the gut microbiome or to indirect effects via modulation of the immune and/or nervous systems.

In summary, additional studies on the role of the gut microbiome in GWI are called for in light of emerging findings that significant enteric dysbiosis has been documented in Veterans with GWI as well as in animal models of this disorder. Each of the major symptom clusters of GWI has been linked individually to alterations in the gut microbiome so it is plausible that an altered gut microbiome could contribute to all major symptoms of this disorder. It is clear that the symptoms of GWI persist long after the toxicants to which military personnel were exposed in the Gulf War (e.g., PER and PB) have been removed from the body. Therefore, emphasis should also be placed on assessing various life-style risk factors for their ability to potentiate and/or extend the chronicity of the symptoms of GWI. There is no medically validated or effective treatment for GWI and if additional substantiation can be gathered for a role for gut microbiome dysbiosis, new and non-invasive therapies that target restoration of the gut microbiome in Veterans with GWI (e.g., probiotics, dietary interventions, fecal transplantation) could be tested as therapies.

## Supplementary information

Supplementary Information.

## Data Availability

The MiSeq 16S rRNA gene sequence data generated in the current study will be made available upon request.
