# Supplementary material for: Effects of a high fat diet on gut microbiome dysbiosis in a mouse model of Gulf War Illness
Source: Sci Rep. 2020 Jun 12;10:9529. doi: 10.1038/s41598-020-66833-w (PMC7293234; doi:10.1038/s41598-020-66833-w)
Supplement: Supplementary file 1 — Supplementary Information. [file 41598_2020_66833_MOESM1_ESM.docx]

**Effects of a high fat diet on gut microbiome dysbiosis in a mouse model of Gulf War Illness**

**Mariana Angoa-Pérez, Branislava Zagorac, Dina M. Francescutti, Andrew D. Winters, Jonathan M. Greenberg, Madison M. Ahmad, Shannon D. Manning, Brian D. Gulbransen, Kevin R. Theis & Donald M. Kuhn**

**Supplementary Information**

Supplementary Figure S1

Supplementary Figure S2

Supplementary Table S1

Supplementary Table S2


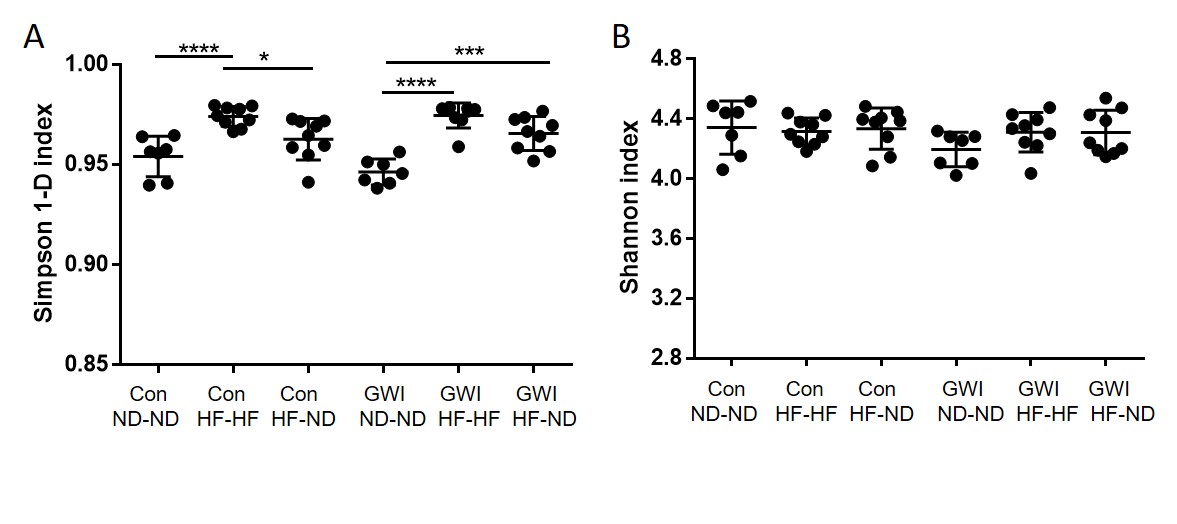


Supplementary Figure S1. Effects of GWI ± HF on α-diversity. Data are presented as Simpson (1-D) index (A) or Shannon index (B) ± SEM, N= 8-9. Con = control; GWI = PER + PB; ND = normal diet; HF = high fat diet. Symbols represent significance levels for the indicated post hoc comparisons as p < *0.05, ***0.001, ****0.0001.

Supplementary Figure S2. Effects of treatments on β-diversity. PCoA showing differences in the similarities of the gut microbiome profiles of the study groups using the Bray-Curtis index (A) and the statistical results of all post hoc statistical tests (B). Con = control; GWI = PER + PB; ND = normal diet; HF = high fat diet.


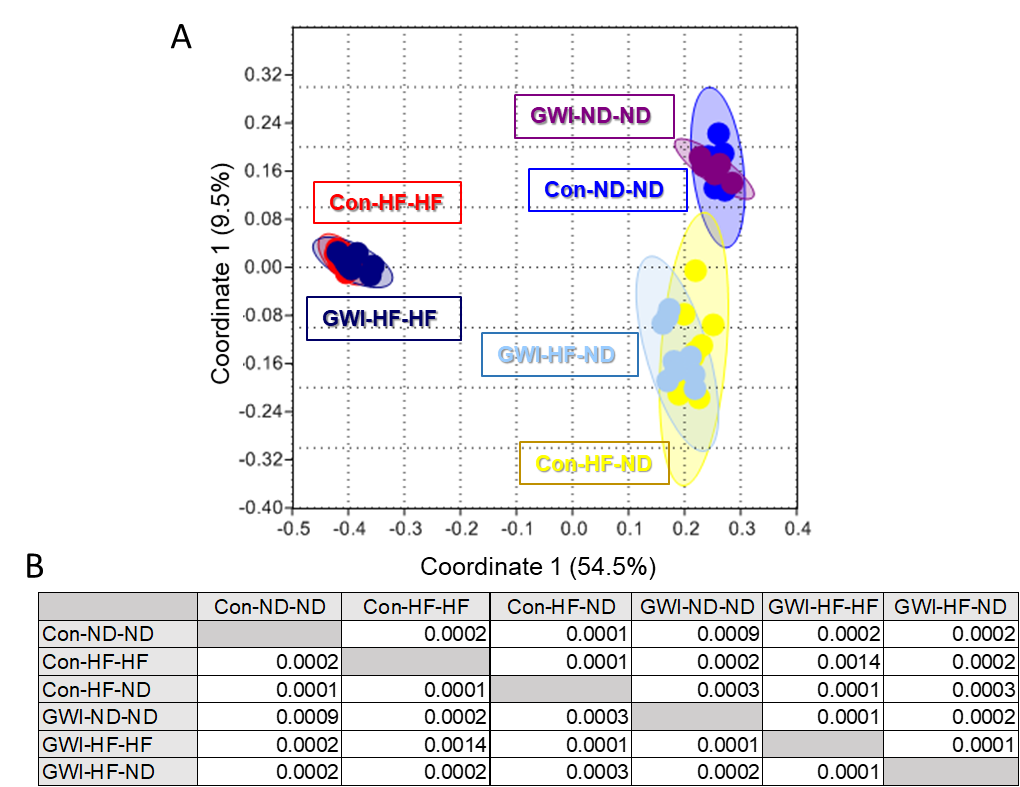


Supplementary Table S1. Statistical results of all post hoc comparisons of β-diversity using the Jaccard index, as illustrated in Figure 3.


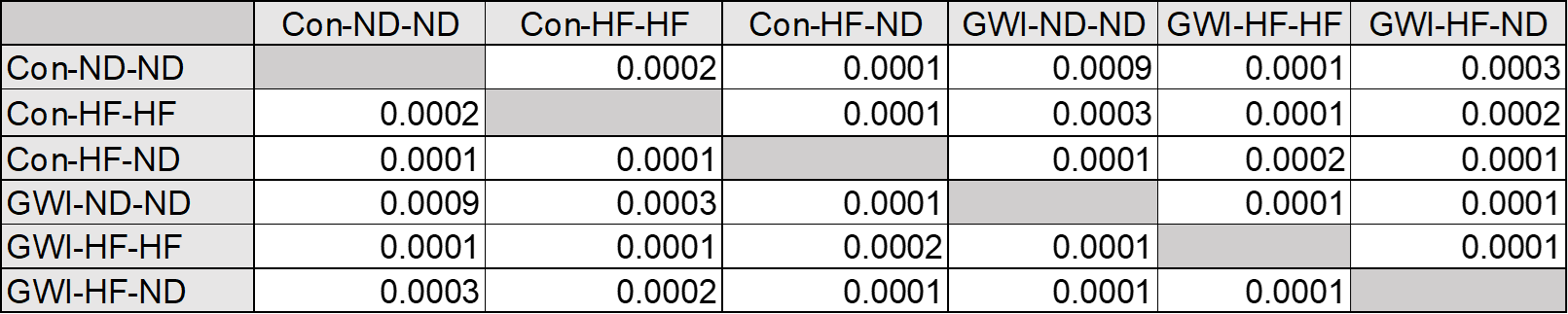

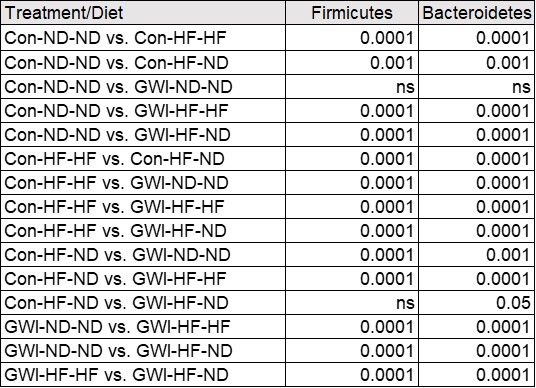


Supplementary Table S2. Statistical results of all post hoc comparisons of the % relative abundance of phyla from Figure 7.
